# Functional Characterization of Melanin Decolorizing Extracellular Peroxidase of *Bjerkandera adusta*

**DOI:** 10.3390/jof7090762

**Published:** 2021-09-15

**Authors:** Jina Baik, Anwesha Purkayastha, Kyung Hye Park, Taek Jin Kang

**Affiliations:** Department of Chemical and Biochemical Engineering, Dongguk University, Seoul 04620, Korea; jbaik124@gmail.com (J.B.); itisanwesha@gmail.com (A.P.); kyung9749@naver.com (K.H.P.)

**Keywords:** *Bjerkandera adusta*, peroxidase, enzymatic melanin decolorization

## Abstract

Melanin pigmentation in the human skin results from complicated cellular mechanisms that remain to be entirely understood. Uneven melanin pigmentation has been counteracted by inhibiting synthesis or transfer of melanin in the skin. Recently, an enzymatic approach has been proposed, wherein the melanin in the skin is decolorized using lignin peroxidase. However, not many enzymes are available for decolorizing melanin; the most studied one is lignin peroxidase derived from a lignin degrading fungus, *Phanerochaete chrysosporium*. Our current study reveals that versatile peroxidase from *Bjerkandera adusta* can decolorize synthetic melanin. Melanin decolorization was found to be dependent on veratryl alcohol and hydrogen peroxide, but not on Mn^2+^. The degree of decolorization reached over 40% in 10 min at 37 °C and a pH of 4.5. Optimized storage conditions were slightly different from those for the reaction; crude enzyme preparation was the most stable at 25 °C at pH 5.5. Since the enzyme rapidly lost its activity at 50 °C, stabilizers were screened. As a result, glycerol, a major component in several cosmetic formulations, was found to be a promising excipient. Our results suggest that *B. adusta* versatile peroxidase can be considered for future cosmetic applications aimed at melanin decolorization.

## 1. Introduction

Melanin is a colored biopolymer synthesized by melanocytes, whose amount and distribution in the human skin greatly affects skin color [1]. Although the complete process is not entirely understood [2], melanin is believed to be synthesized in the melanosome and then transferred to neighboring keratinocytes, which protect their nuclei from harmful ultraviolet radiation [3]. Therefore, the timely synthesis and subsequent translocation of this pigment molecule is crucial to ensure the safety of the human skin cell DNA from sunlight. However, abnormally increased amounts of melanin released in localized spots (hyperpigmentation) leads to an uneven and dark skin tone. Based on experience, it is widely understood that this uneven dark skin tone is associated not only with sun exposure but also with aging. An even skin tone is widely perceived as an indicator of beauty and youth [4], which makes hyperpigmentation a major cosmetic target.

Treating hyperpigmentation to obtain an even skin tone requires inhibiting either the overactive synthesis of melanin [5,6,7,8] or its transport process [9,10]. Currently, the most popularly prescribed skin lightening agent is hydroquinone, which inhibits melanin synthesis [1,6,11]. Although hydroquinone has being used for several decades, its “generally regarded as safe and effective” status is being reconsidered by the US Food and Drug Administration in over-the-counter drugs (https://www.fda.gov/drugs/status-otc-rulemakings/rulemaking-history-otc-skin-bleaching-drug-products#Labeling, accessed on 13 September 2021). In fact, many adverse effects such as erythema, irritation, and stinging have been reported due to its use [1]. On the other hand, topical application of melanin-degrading enzymes can be considered to achieve an even and lighter skin tone without perturbing melanin’s biosynthetic pathways [12,13,14,15,16,17,18]. Notably, lignin peroxidase (LiP) derived from *Phanerochaete chrysosporium* has demonstrated skin lightening activity in a randomized study showing a greater potency than 2% hydroquinone [19]. Cosmetic formulations containing LiP have also been proven to be efficacious in treating melasma patients [20].

LiP is a heme-containing enzyme and can oxidatively degrade lignin, which is an extremely recalcitrant biomass. Melanin is also an electron-rich durable biopolymer that structurally resembles lignin; therefore, it can be the substrate for LiP [21]. *P. chrysosporium* LiP, either as a fermentation product of the mold [17,21] or as a recombinant protein expressed in *Escherichia coli* [16], has been extensively studied. Diverse white rot fungi are known to produce various lignin degrading enzymes; therefore, testing their melanin decolorizing activities is imperative. Here, we report that the liquid culture of *Bjerkandera adusta* produced peroxidase capable of decolorizing melanin in the presence of veratryl alcohol and hydrogen peroxide. We also investigated some basic characteristics of the peroxidase preparation to confirm its potential for future cosmetic applications.

## 2. Materials and Methods

### 2.1. Materials

All chemicals including potato dextrose broth and synthetic melanin were purchased from Merck (Darmstadt, Germany) and used without any further purification. All fungal strains were obtained from the Korean mushroom collection maintained by the Seoul National University, Korea.

### 2.2. Fungal Cell Cultivation

*B. adusta* KMRB15062613 cells were cultivated on potato dextrose agar (PDA) plates for 2 weeks at 25 °C. The PDA plates were overlayed with 1% (*v*/*v*) aqueous Tween-80, and the scraped conidial suspension was filtered through a sterile gauze to obtain the spore suspension. For liquid cultures, 1.0 × 10^9^ spores L^−1^ were inoculated into 50 mL of sterilized culture media in 250-mL Erlenmeyer flasks. The liquid growth medium (1 L) comprised 20 mM acetate buffer (pH 4.5), 6 g of glucose, 0.2 g of ammonium tartrate, 2 g of KH_2_PO_4_, 0.1517 g of nitrilotriacetate trisodium salt, 0.71 g of MgSO_4_, 0.07 g of NaCl, 0.007 g of FeSO_4_·7H_2_O, 0.007 g of ZnSO_4_·7H_2_O, 0.0011 g of CuSO_4_·5H_2_O, 0.0007 g of AlK(SO_4_)_2_·12H_2_O, 0.0007 g of H_3_BO_3_, 0.0007 g of Na_2_MoO_4_·2H_2_O, 0.0132 g of CaCl_2_, 0.0126 g of CoCl_2_·6H_2_O, 0.001 g of thiamine, 0.54 mL of Tween-80, and 4 mM veratryl alcohol. Cultures were maintained at 30 °C for 30 days with agitation at 100 rpm.

### 2.3. Crude Enzyme Preparation

Enzyme solutions were prepared through sequential filtrations. First, cultures were filtered through 40-μm filters (SPL, Pocheon, Korea); subsequently, the filtrate was stored at −80 °C for at least 12 h to cryoprecipitate the sticky matter that could be conveniently removed by one more round of 40-μm filtration. The sticky matters were not proteinaceous as revealed by sodium dodecyl sulfate polyacrylamide gel electrophoresis (SDS-PAGE) and were presumed to be predominantly carbohydrates based on the literature [12]. Spores were removed by subsequent tandem microfiltrations with 0.45-μm and 0.22-μm filters (Corning, Glendale, AZ, USA). Collected filtrates were concentrated by ultrafiltration with a 10-kDa molecular weight cut-off (Amicon, Merck, Darmstadt, Germany). As described in the text, the Britton–Robinson (BR) buffer with varying pHs was used for certain preparations, and all preparations were stored at −80 °C until use.

### 2.4. Activity Assays

Peroxidase activities of the crude enzyme preparations were measured using veratryl alcohol or melanin as the substrate in the presence of hydrogen peroxide. Activities are represented in units (U) or degree of melanin decolorization using veratryl alcohol or synthetic melanin as substrates, respectively. For veratryl alcohol oxidation, 1 U of enzyme activity was defined as the amount of enzyme preparation required to oxidize 1 μmol of veratryl alcohol into veratraldehyde per min. Degree of melanin decolorization was defined as the percentage reduction in melanin concentration compared to the initial level during the reaction.

When veratryl alcohol was used as the substrate, the assay mixtures comprised BR buffer (pH 3.0), 2 mM veratryl alcohol, 0.25 mM H_2_O_2_, and 10% (*v*/*v*) enzyme solution; unless specified otherwise, all reactions were carried out at 37 °C. Veratraldehyde generation was traced by continuously monitoring absorbance changes at 310 nm (ε = 9300 M^−1^cm^−1^) for the first 1−2 min of the reaction.

When melanin was used as the substrate, the peroxidase reaction was conducted in the presence of BR buffer (pH 4.5), 2 mM veratryl alcohol, 0.25 mM H_2_O_2_, 0.1 mg mL^−1^ synthetic melanin, and 0.12 U mL^−1^ enzyme preparation. In order to assess the enzyme activities in serum formulation (COSMAX, Seongnam, Korea), the reaction mixtures were mixed with equal volumes of either the serum formulation or water. Typical reactions were conducted in a microplate reader at 37 °C for 1 h with regular spectrophotometric absorbance measurements at 475 nm to detect melanin.

### 2.5. Effects of pH and Temperature

In order to investigate the effects of pH on peroxidase activities, the reaction pH was modulated using the BR. The reactions were carried out at 37 °C. In order to investigate the effects of temperature, solutions containing veratryl alcohol, enzyme preparations, and BR buffer (pH 3.0) were pre-incubated for 5 min at various temperatures (27–45 °C) for equilibration. The reactions were initiated by addition of H_2_O_2_.

### 2.6. Stability Tests

The thermal stabilities of the enzyme preparations were evaluated through melanin assays preceded by pre-incubation at various temperatures for 12 h. Various buffer pHs were also tested before pre-incubating the enzyme preparations at a designated temperature. In order to investigate the effects of protective excipients on the storage stability of the enzyme preparations at 50 °C, CaCl_2_ (0.8 mM), tryptophane (0.8 mM), or glycerol (20% *v*/*v*) were added during the pre-incubation. These excipient concentrations were reduced to 0.2 mM (CaCl_2_ and tryptophane) or 5% (*v*/*v*), respectively, in the melanin decolorization reactions. Equal concentrations of excipients were ensured in the control reactions to identify their possible inhibitory effects on the melanin decolorization reaction.

## 3. Results

### 3.1. Preparation of the Peroxidase Solution from B. adusta Mycelial Cultures

**Figure 1:**
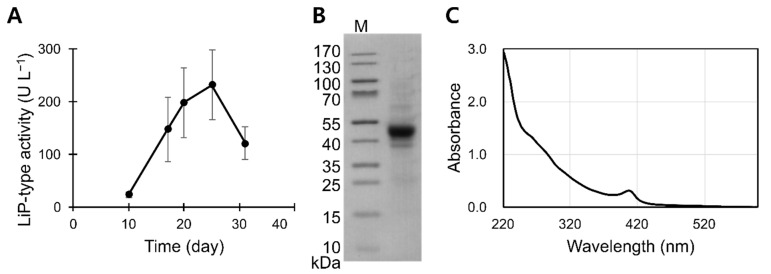
Preparation of POX_Ba. Extracellular titers of LiP-type activities were measured from biological triplicates (**A**). Error bars are standard deviations. SDS-PAGE analysis revealed one major band in concentrated POX_Ba (**B**). M, molecular weight marker. Concentrated POX_Ba exhibited a Soret peak at 407 nm (**C**), which strongly suggested that POX_Ba contained a heme structure. The spectrum was obtained using ~0.3 mg mL^−1^ POX_Ba in a BR buffer (pH 5.5).

Initially, we screened six white rot fungi strains for extracellular peroxidase activities using veratryl alcohol and hydrogen peroxide as co-substrates. Mycelial strains that were tested included *Trametes versicolor*, *Ganoderma lucidum*, and *B. adusta*. Of these, *B. adusta* consistently exhibited notable peroxidase activities in our culture conditions. Typically, it took 25−28 days to maximize veratryl alcohol oxidizing activities in the culture broths of *B. adusta* (Figure 1A), which was significantly longer than the previously reported 5 days required to maximize LiP activity in *P. chrysosporium* cultures [22]. Throughout this article, this Mn^2+^-independent veratryl alcohol oxidizing activity is referred to as ‘LiP-type activity’.

Immobilizing *B. adusta* cells on polyurethane foam blocks maximized the LiP activity within 10 days of culturing [23]. However, without immobilization, the cells exhibited no LiP activity in the same medium. Therefore, further rigorous optimizations of the growth media or culturing conditions could yield higher productivity in future studies. Nonetheless, flask cultures yielded veratryl alcohol oxidizing activity of over 230 U L^−1^. Upon serial filtration of the cultures, only one major protein band with an apparent molecular weight of 45 kDa was revealed by the SDS-PAGE analysis (Figure 1B). The apparent molecular weight indicates that the protein band could represent LiP, manganese peroxidase (MnP), versatile peroxidase (VP), or their combination [24,25,26]. Strong absorption of 407 nm light confirmed that the preparation includes heme-containing peroxidase (Figure 1C).

Using the highly concentrated enzyme solution, we next attempted to characterize some enzymatic properties of the peroxidase preparation, which is hereafter referred to as POX_Ba.

### 3.2. LiP-Type Activity in POX_Ba Is Likely from VP

**Figure 2:**
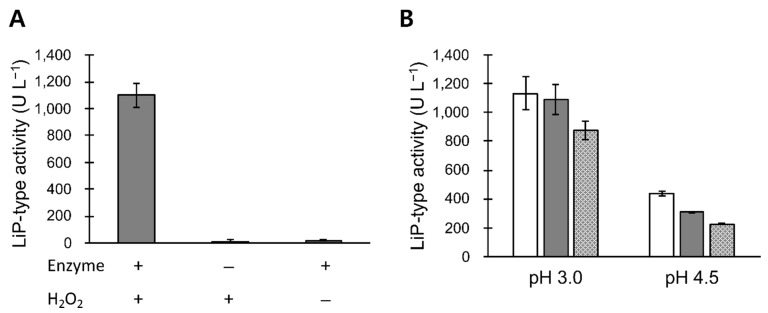
(**A**) Characterization of POX_Ba. Hydrogen peroxide was required for POX_Ba to oxidize veratryl alcohol (LiP-type activity), which indicated the absence of an aryl-alcohol oxidase. (**B**) The LiP-type activity of POX_Ba was negatively affected in a concentration-dependent manner by Mn^2+^, the primary substrate of MnP. White, grey, and patterned bars contained 0, 0.3, and 2 mM Mn^2+^, respectively. The inhibitory effect of Mn^2+^ was more significant at a pH of 4.5 (optimal for MnP-type activity of VP) compared to at a pH of 3.0 (reportedly optimal for the LiP-type activity of VP), which suggested competition between the LiP- and MnP-type activities of the same enzyme (likely VP). Error bars, standard deviations for triplicated reactions.

The concentrated *B. adusta* culture broth was able to oxidize veratryl alcohol only in the presence of hydrogen peroxide (Figure 2A), indicating that POX_Ba was not an aryl-alcohol oxidase. Addition of 0.3 or 2 mM Mn^2+^ to the reaction inhibited the LiP-type activity in a concentration- and pH-dependent manner (Figure 2B). At a pH of 3, the LiP-type activity of POX_Ba was only slightly decreased by addition of 0.3 mM Mn^2+^, whereas 2 mM Mn^2+^ inhibited the activity by 23%. At a higher pH level of 4.5, 2 mM Mn^2+^ more significantly inhibited the LiP-type activity (~50%) of POX_Ba. This suggested that POX_Ba was more likely to contain VP than LiP because similar properties have been reported for VP purified from *B. adusta* [27,28]. Previous studies have reported that the MnP-type activity of VP (oxidation of Mn^2+^ to Mn^3+^) is optimized at a pH of 4.5, whereas its LiP-type activity is the highest at a pH of 3. Although VP can oxidize both veratryl alcohol and Mn^2+^, the catalytic cycle involves the oxidation of a heme as the first step, which allows VP to only oxidize one. Therefore, it was proposed that the MnP-type activity of VP outcompetes the LiP-type activity in the presence of Mn^2+^, the primary substrate of MnP, at a pH of 4.5 [27,28]. However, we do not rule out the possibility that our preparation (POX_Ba) contained several types of lignin degrading enzymes. Instead of purifying certain specific enzymes from the preparation, we intended to test the use of the entire preparation as a cosmetic active ingredient in the absence of Mn^2+^.

**Figure 3:**
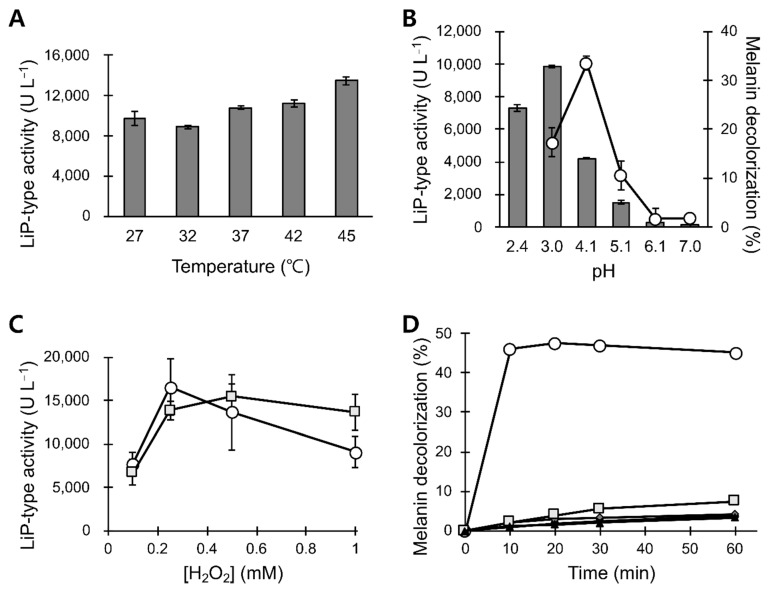
Characterization of POX_Ba reactions. (**A**) Effect of reaction temperature on the LiP-type activity of POX_Ba. All reactions were initiated by the addition of hydrogen peroxide to the pre-equilibrated reaction at designated temperatures. (**B**) Effects of reaction pHs on the LiP-type and melanin decolorizing activity of POX_Ba. Grey bars, LiP-type activities; circles in a line graph, % melanin degradation. (**C**) Effect of hydrogen peroxide concentration on the LiP-type activity of POX_Ba. Reactions were conducted in the presence of 2 mM (circle) or 4 mM (square) veratryl alcohol. (**D**) Melanin decolorization required the presence of POX_Ba, H_2_O_2_, and veratryl alcohol (circle). When the reactions were deficient in veratryl alcohol (square), H_2_O_2_ (diamond), or POX_Ba (triangle), no significant decolorization was observed. In the presence of only hydrogen peroxide, POX_Ba could decolorize melanin to less than 8% (square). Error bars, standard deviations from triplicated reactions.

In the temperature range of 27–45 °C, POX_Ba exhibited slightly increased LiP-type activities at higher temperatures when the reaction was buffered at a pH of 3 (Figure 3A). Interestingly, both *P. chrysosporium* LiP [29] and *B. adusta* VP [28] exhibit higher optimal reaction temperatures as the reaction pHs are increased. Therefore, optimal reaction temperature can only be determined for a specific pH level. Considering the normal body temperature, we further characterized POX_Ba at a fixed temperature of 37 °C. Similar to other lignin degrading peroxidases, POX_Ba was active only in acidic conditions with its optimal activity at a pH of 3.0 when veratryl alcohol was used as a substrate (Figure 3B). At a pH of 3.0, the LiP-type activity of POX_Ba was optimal upon addition of 250 μM H_2_O_2_ when 2 mM veratryl alcohol was used (Figure 3C). Upon increasing the concentration of veratryl alcohol to 4 mM, POX_Ba seemed to tolerate higher concentrations of H_2_O_2_; however, the maximum LiP-type activity did not change (Figure 3C).

As described above, the highest LiP-type activity of POX_Ba was achieved when the reaction was buffered at a pH of 3.0 (Figure 3B). The optimal pH level to decolorize synthetic melanin was slightly higher (4.1 to 3.0, shown in Figure 3B), even though efficient melanin decolorization required veratryl alcohol as a mediator for the reaction (Figure 3D). Considering the fact that low pH levels are beneficial to the skin [30], demonstrating melanin decolorization at pH levels 4–4.5 seemed reasonable. Melanin decolorization of 40–50% was observed within 10 min in the presence of 250 μM H_2_O_2_ and 2 mM veratryl alcohol at 37 °C and a pH of 4.5.

As previously reported, *P. chrysosporium* LiP exhibits higher specific activities toward veratryl alcohol with H_2_O_2_ concentrations much higher than 250 μM; however, the high H_2_O_2_ concentration tends to deactivate the enzyme [16,21]. Periodically supplementing H_2_O_2_ to the reaction or generating it in situ using glucose and glucose oxidase thereby prolonged the melanin decolorization reaction in the mentioned works. We also attempted to supplement the melanin decolorization reaction with glucose oxidase and glucose; however, that did not enhance melanin decolorization (data not shown). Next, we evaluated the storage stability of POX_Ba.

### 3.3. pH and Temperature Stability of POX_Ba

**Figure 4:**
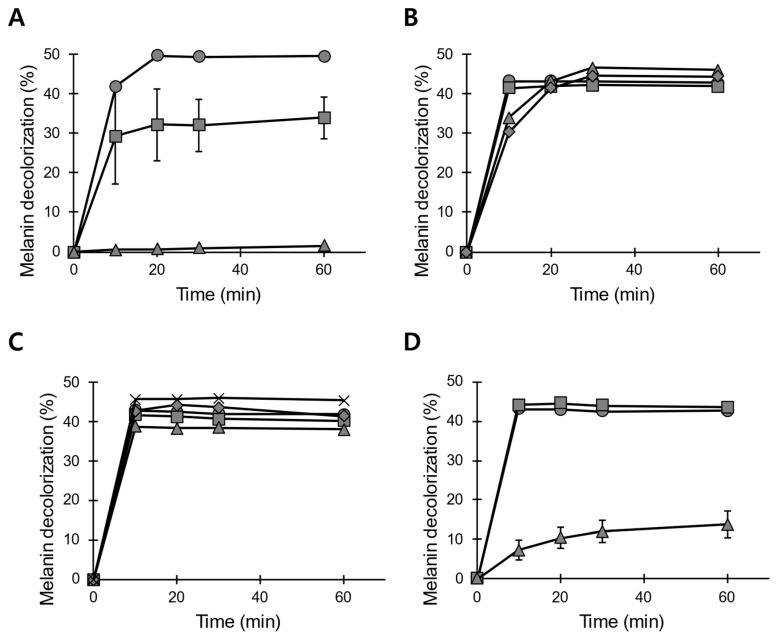
Effects of pH and temperature on the storage stability of POX_Ba. Melanin decolorizing activities of POX_Ba were assessed after 0 (circles), 1 (squares), 7 (triangles), 14 (diamonds), and 28 (×) days of preincubation at 25 °C. Before the preincubation, POX_Ba was buffer exchanged to none (**A**), pH 4.5 (**B**), pH 5.5 (**C**), or pH 6.5 (**D**) using BR buffers. Results represent averages of three independent preincubation-reaction samples, and the error bars represent their standard deviations. Samples without error bars exhibited relative standard deviations (standard deviation divided by the average) of lower than 10%.

An important attribute of ‘good enzymes’ is the stability both during the reaction and storage; the conditions for optimal storage and optimal activity do not always coincide. POX_Ba, which is a mere concentrate of high molecular weight species derived from the *B. adusta* culture broth, exhibited a pH of ~6. When stored at 25 °C, POX_Ba started losing its melanin decolorizing activity within a day, with it being completely lost after 2 days of incubation (Figure 4A). In order to increase the storage stability of POX_Ba, the pH levels of the crude preparation were changed to 4.5, 5.5, 6.5, and 7.5 using diafiltration; the resulting buffer-exchanged preparations were designated as POX_Ba (pH 4.5), etc. Upon incubation at 25 °C, POX_Ba (pH 5.5) did not lose its melanin decolorizing activity for at least 4 weeks (Figure 4C). POX_Ba (pH 4.5) also retained complete activity for at least 2 weeks with melanin decolorization reaction times of 30 min or longer; however, a slight decrease in its initial activity was observed (Figure 4B). Although POX_Ba (pH 6.5) lost most of its activity (~85%) upon a 7-day incubation at 25 °C, the retained activity still significantly decolorized melanin compared to that of POX_Ba without the buffer exchange (Figure 4A vs. Figure 4D). It is possible that small molecules in the culture broth make the enzymes more vulnerable to thermal deactivation. POX_Ba (pH 7.5) completely lost its activity after incubation at 25 °C for 1 day.

**Figure 5:**
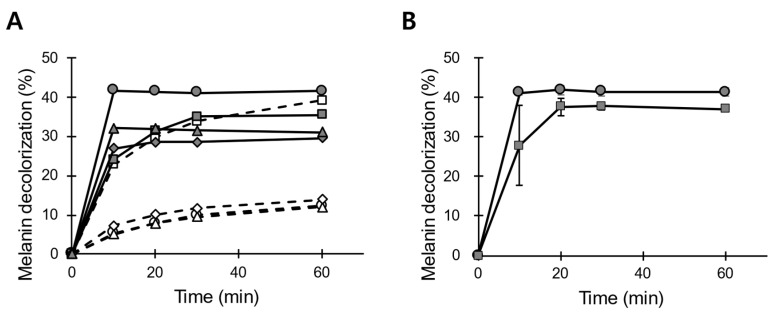
Effects of excipients on the storage stability of POX_Ba. (**A**) POX_Ba (pH 5.5) was either preincubated for 12 h at 50 °C (dashed lines) or freshly taken from −80 °C (solid lines) before being added to the melanin decolorization reaction. Excipients were included in the preincubations or added to the reactions with fresh enzyme solution to yield the same final concentrations. Circle, no excipient; square, glycerol; triangle, tryptophane; diamond, CaCl_2_. Results represent averages of triplicated reactions, and error bars are not shown for easier data interpretation. (**B**) Melanin decolorization upon 1:1 dilution with water (circle) or serum formulation containing polyols (square).

The storage and handling of POX_Ba strictly at 25 °C during the manufacturing processes is not realistic; therefore, we elevated the incubation temperature to 50 °C. Upon preincubation for 12 h at 50 °C, POX_Ba (pH 5.5) exhibited a melanin decolorization activity of only ~13% even with a reaction time of 1 h, which was about 70% lower than that of the untreated enzyme solution (Figure 5). POX_Ba (pH 5.5) was chosen for these analyses because in addition to a pH of 5.5 being the best for the storage of POX_Ba at 25 °C (Figure 4), there was a loss of the enzyme (>20% presumably as precipitates) in other buffer-exchanged solutions with different pH levels (data not shown). The degree of melanin decolorization in the initial phase (10 min) was even poorer (~5%), displaying a relative activity of 13% to that of the untreated enzyme solution. Then, thermostabilizing excipients, Ca^2+^, tryptophane, or glycerol, were added to POX_Ba. Many heme-containing peroxidases have been reported to properly fold only in the presence of calcium ion(s), and the loss of Ca^2+^ has been shown to lead to protein inactivation. For example, the thermal inactivation of *P. chrysosporium* LiP has been shown to result from the loss of Ca^2+^ from its binding sites; supplementation of Ca^2+^ during storage has been shown to greatly slow down the inactivation rate [31]. *B. adusta* VP has also been reported to lose its activity upon the depletion of Ca^2+^ [32]. On the other hand, tryptophane has been shown to protect LiP from the oxidative damage exerted by fermentative H_2_O_2_ [33]. Both Ca^2+^ and tryptophane, however, failed to protect POX_Ba (pH 5.5) from thermal inactivation (Figure 5). More importantly, both excipients inhibited the melanin decolorization reaction to some degree (grey diamond and triangle in Figure 5). Notably, glycerol, a well-known protein-stabilizing polyol, greatly stabilized POX_Ba (pH 5.5) against heat-inactivation, even though there was a slight decrease in the initial melanin decolorization rate (squares in Figure 5A).

The reason for glycerol slowing down the melanin decolorization reaction is still unclear. However, this could potentially be problematic in using POX_Ba as a cosmetic ingredient because glycerol is widely used in many cosmetic formulations. In order to address this concern, we checked melanin decolorization in the presence of a general water-based serum formulation. Basically, the melanin decolorization reactions were diluted with water or the serum, and, as can be seen in the Figure 5B, POX_Ba (pH 5.5) could decolorize up to ~40% of melanin tested within 20 min at 37 °C in both cases. However, the reaction diluted with the serum was slightly less efficient than that diluted with water, especially in the initial phase (10 min in Figure 5B). This suggests that ingredients in the serum, including glycerol, could have protected melanin from being oxidatively decolorized to some extent, but not entirely. In contrast, guar gum, which is used as another protein stabilizing polyol and also as a thickener in the serum formulation, severely inhibited the melanin decolorization reaction (data not shown). Whether polyols generally inhibit the melanin decolorizing activity of POX_Ba is still elusive.

## 4. Discussion

The identification of a lignin degrading enzyme, LiP, that could decolorize melanin, has resulted in the investigation of its cosmetic applications [19,20,21]. In this study, liquid cultures of *B. adusta* produced peroxidase, demonstrating the melanin decolorizing activity. The presence of Mn^2+^ in the medium has been reported to reduce LiP production while inducing MnP production in another white rot fungi, *P. chrysosporium* [22,34]; therefore, we initially expected the omission of a manganese salt from the minimal salt medium to yield higher titers of LiP. As another potent lignin degrading enzyme, MnP, can also decolorize melanin [13,15]; however, its dependence on Mn^2+^ makes it undesirable for cosmetic applications [12]. The culture conditions used here presumably produced VP as the major product (Figure 1B and Figure 2B). In fact, unlike other white rot fungi [35,36], *B. adusta* cultures exhibited Mn^2+^-oxidizing activities in the absence of a manganese salt, which was later determined to be from VP and not from MnP [27].

VP has both LiP and MnP activities; therefore, it is not surprising that it decolorizes melanin. However, to the best of our knowledge, VP has never been reported to be used in melanin decolorization. Our results suggest the possibility of using VP as a cosmetic ingredient. Whether the observed melanin decolorization was because of VP alone or in combination with other peroxidases, such as LiP, still needs to be determined through future studies. The primary goal of this study was to determine whether the culture broth from *B. adusta* could be used to decolorize melanin without requiring extensive, pharmaceutical-level purification. Our results demonstrated that POX_Ba is active at body temperatures close to the pH level of the healthy skin and could decolorize ~40% of synthetic melanin in 10 min (Figure 3).

Examining the storage stabilities revealed that the pH level of the storage medium was important in both stabilizing the activity and preparing the enzyme solution. At an optimized pH of 5.5, which was slightly higher than that for the reaction, the decolorizing activity was not compromised even after 4 weeks of incubation at 25 °C (Figure 3 and Figure 4). When glycerol was used as an excipient, POX_Ba (pH 5.5) was substantially stable at an elevated temperature (50 °C), even though it retarded the melanin decolorization reaction. This prompted us to test POX_Ba activity in a serum formulation containing glycerol and other polyols, which exhibited a slightly decreased but significant melanin decolorization in 20 min (Figure 5). Therefore, we expect that POX_Ba will be found useful in cosmetic applications aimed at lightening skin tone through on-site melanin decolorization.

## Data Availability

Not applicable.
